# Envisioning the future of the Jornal Brasileiro de Pneumologia

**DOI:** 10.36416/1806-3756/e20250425

**Published:** 2025-11-19

**Authors:** Marcia Margaret Menezes Pizzichini, Denise Rossato Silva

**Affiliations:** 1 Editor-in-Chief of the Jornal Brasileiro de Pneumologia; 2 Vice-Editor-in-Chief of the Jornal Brasileiro de Pneumologia

The golden jubilee of the *Jornal Brasileiro de Pneumologia* (JBP) marks a significant achievement for nearly 4,000 members of the *Sociedade Brasileira de Pneumologia e Tisiologia* (SBPT, Brazilian Thoracic Association). To celebrate this milestone, the JBP is publishing four editorials reflecting on its history and outlining our aspirations for the future.

The journey of the JBP has been characterized by many unexpected developments, revealing a distinct trend toward consolidating its success. For example, over the past decade, we have made considerable advances in the quality of our publications, as evidenced by our improved rankings from the National Council for Research (Qualis-CNPq) and international recognition from prestigious organizations such as Scimago Journal & Country Rank, Journal Citation Report (JCR), and Scopus. In 2024, the JBP achieved a notable milestone by ranking in the Q2 quadrant among 105 international scientific journals in Respiratory Medicine, securing the 42nd position, a journal impact factor of 3.0, and an H-index of 52. Our citation index, as reported by Scopus, currently stands at 4.0, underscoring the relevance of our publications over the past five years, with 78% of original articles cited.

The JBP ranks 9th among 391 journals across all disciplines in Brazil. Additionally, our publications have considerably attracted significant attention, averaging approximately 6,000 views per paper. Notably, the 2020 Brazilian Thoracic Association recommendations for asthma management have achieved an impressive 238,532 views to date, demonstrating our potential for growth and broader reach beyond our Association and Brazil.

Despite such promising advancements, the current and former Editors of the JBP, along with our team of Associate Editors, recognize that we are only at the beginning of our journey towards greater international recognition and visibility. We envision a future when the JBP transcends its regional identity by adopting a name in English. This change aims to attract scientific manuscripts from around the globe and to facilitate the dissemination of free-of-charge translational research in respiratory and pulmonary health.

We acknowledge that this transformation may initially result in a decline in our JCR ranking and citation counts for a period of three years. However, we are fully committed to this investment in the long-term growth of the Journal. It is important to clarify that this does not reflect a decrease in the quality of our publications; on the contrary, it is the result of transitioning to a new name, which may split citations between “*Jornal Brasileiro de Pneumologia*” and “the new title to be chosen.”

As we embark on this transformative journey, we want to emphasize our unwavering commitment to maintaining the high standards of scholarly excellence that have defined the JBP for the past fifty years. Our rich history is an indication to the dedication of our editorial team and reviewers, as well as the invaluable contributions of our authors, who have consistently expanded the frontiers of knowledge in pulmonary medicine. This collaborative spirit has fostered an environment conducive to innovation and rigorous scientific inquiry. To strengthen our international reach further, we will be implementing strategies to enhance the visibility of our Journal. These include increased promotional efforts at international conferences, partnerships with global research networks, and an expanded presence on digital platforms. By doing so, we aim not only to attract more submissions from around the globe but also to ensure that our research findings reach a wider audience, thereby maximizing the impact of our work.

Moreover, we are committed to fostering an inclusive platform that welcomes diverse perspectives and encourages international collaborations. This commitment aligns with our mission to advance research that addresses key challenges in respiratory health, particularly in underrepresented populations and settings. We believe that a more inclusive approach will enrich the quality of our publications and better reflect the global nature of research today.

To support our emerging vision, we are also exploring the implementation of new initiatives, such as special issues focused on cutting-edge topics in pulmonology, as well as webinars and workshops aimed at educating authors on best practices for manuscript submission and publication. Our goal is to create a supportive ecosystem that empowers researchers at all stages of their careers, enhancing both the quality and quantity of contributions to our Journal.

In conclusion, as we celebrate this golden jubilee, we stand at a pivotal moment in the evolution of the JBP. With unwavering commitment and a clear vision for the future, we are excited to take the next steps in order to elevate our journal to new heights. We invite all members of the SBPT and the global research community to join us on this journey, as we collectively strive to advance knowledge and improve the health outcomes of individuals affected by respiratory diseases. Together, we will build a stronger, more visible, and impactful JBP for the years to come.


Marcia Margaret Menezes Pizzichini Editor-in-Chief of the Jornal Brasileiro de Pneumologia
Denise Rossato Silva Vice-Editor-in-Chief of the Jornal Brasileiro de Pneumologia


